# Tailorable Synthesis of Highly Oxidized Graphene Oxides via an Environmentally-Friendly Electrochemical Process

**DOI:** 10.3390/nano10020239

**Published:** 2020-01-29

**Authors:** Ana María Díez-Pascual, Carlos Sainz-Urruela, Cristina Vallés, Soledad Vera-López, María Paz San Andrés

**Affiliations:** 1 Department of Analytical Chemistry, Physical Chemistry and Chemical Engineering, Faculty of Sciences, University of Alcalá, Alcalá de Henares, 28805 Madrid, Spainsoledad.vera@uah.es (S.V.-L.); mpaz.sanandres@uah.es (M.P.S.); 2 Institute of Chemistry Research, “Andrés M. del Río” (IQAR), University of Alcalá, Ctra. Madrid- Barcelona Km. 33.6, Alcalá de Henares, 28805 Madrid, Spain; 3 Department of Materials and National Graphene Institute, University of Manchester, Oxford Road, Manchester M13 9PL, UK; cristina.valles@manchester.ac.uk

**Keywords:** graphene oxide, electrochemical synthesis, oxidation level, exfoliation degree, morphology, interlayer spacing, surface defects, electrical resistance

## Abstract

Graphene oxide (GO) is an attractive alternative to graphene for many applications due to its captivating optical, chemical, and electrical characteristics. In this work, GO powders with a different amount of surface groups were synthesized from graphite via an electrochemical two-stage process. Many synthesis conditions were tried to maximize the oxidation level, and comprehensive characterization of the resulting samples was carried out via elemental analysis, microscopies (TEM, SEM, AFM), X-ray diffraction, FT-IR and Raman spectroscopies as well as electrical resistance measurements. SEM and TEM images corroborate that the electrochemical process used herein preserves the integrity of the graphene flakes, enabling to obtain large, uniform and well exfoliated GO sheets. The GOs display a wide range of C/O ratios, determined by the voltage and time of each stage as well as the electrolyte concentration, and an unprecedented minimum C/O value was obtained for the optimal conditions. FT-IR evidences strong intermolecular interactions between neighbouring oxygenated groups. The intensity ratio of D/G bands in the Raman spectra is high for samples prepared using concentrated H_2_SO_4_ as an electrolyte, indicative of many defects. Furthermore, these GOs exhibit smaller interlayer spacing than that expected according to their oxygen content, which suggests predominant oxidation on the flake edges. Results point out that the electrical resistance is conditioned mostly by the interlayer distance and not simply by the C/O ratio. The tuning of the oxidation level is useful for the design of GOs with tailorable structural, electrical, optical, mechanical, and thermal properties.

## 1. Introduction

**Scheme 1:**
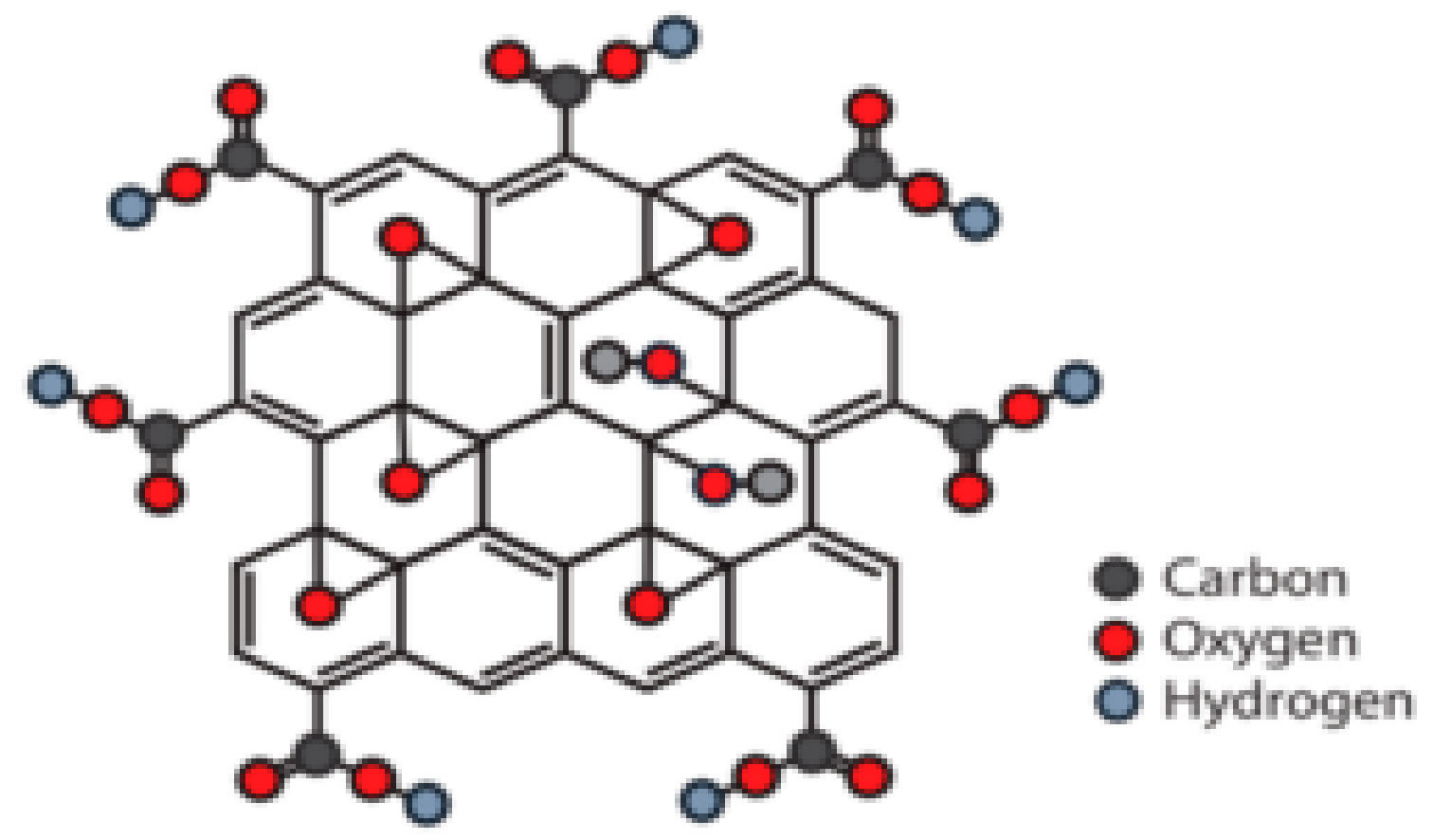
Representation of the chemical structure of graphene oxide (GO).

Graphene oxide (GO), which is the oxidized form of graphene, is currently attracting a lot of interest due to its unique chemical, optical, and electronic properties that make it suitable for a broad range of uses including supercapacitors, solar cells, fuel cells, lithium batteries, biomedicine, polymer nanocomposites, and more [1,2,3,4,5,6]. It presents several surface oxygen-containing groups, mainly COOH moieties on the layer edges as well as C-O-C and OH on the basal planes (Scheme 1). Furthermore, the hydroxyl groups can bond to form epoxy groups, which leads to a distorted tetrahedron with four carbon atoms on the six-membered ring of the carbon plane. This provokes the flat graphene network to bend. Therefore, some characteristics of GO differ from those of graphene. The oxygenated groups in GO expand the interlayer distance, which boosts its skill to retain substances. The attached groups and lattice defects alter the electronic structure and act as scattering centers that reduce the electron mobility. Hence, it is typically an insulating material. Furthermore, it presents biocompatibility, surface functionalization capability, amphiphilicity, and aqueous processability [7]. Thus, after sonication in aqueous solutions, it straightforwardly exfoliates, which leads to stable colloidal suspensions [8]. This can be used for the preparation of transparent electrodes. The presence of oxygen functionalities also makes GO a good candidate for environmentally-related applications such as wastewater treatment and water purification to remove both inorganic and organic pollutants including hormones or antibiotics [9]. GO is also largely used for the synthesis of a number of derivatives to be used in environmental and energy-related applications [10].

The adjustment of the oxidation level would be useful for the design of GOs with tailorable structural, electrical, mechanical, thermal, and optical properties. For instance, the energy band gap of GO increases linearly while decreasing the C/O ratio [11], which provides an efficient way to tune the optical properties of graphene oxide-based materials. On the other hand, as the degree of oxidation increases, both the Young’s modulus and mechanical strength will likely decrease monotonically due to the breaking of the sp^2^ carbon network and lowering of the energetic stability for the ordered GO [12]. However, the increase in the oxidation level of GO could be beneficial for improving the mechanical properties of nanocomposites [13], especially in the case of matrices involving oxygenated groups like chitosan [14] or polyamide [15]. In such cases, the mechanical strength was enhanced with the increase in the oxidation degree due to the stronger H-bonding interactions between the GO sheets, the polymeric matrices, and the more extended chemical interphase. Other properties including the heat capacity, thermal conductivity, and specific capacitance could also be tailored by modifying the oxidation degree [16,17].

In the 19th century, graphite oxide (also known as graphite acid) was first synthesized from graphite through oxidation using KClO_3_ in fuming HNO_3_. Since then, a lot of effort has been made in order to obtain more oxidized and exfoliated graphite oxide. Four main approaches have been described for the synthesis of GO [18]: Staudenmaier, Hofmann, Brodie, and Hummers. Both Brodie and Staudenmaier routes use KClO_3_ and HNO_3_, and present an explosion hazard as well as evolution of dangerous and carcinogenic gases such as NOx, ClO-, and ClO_2_. The most widely employed is the Hummers’ method, which comprises the mixing of KMnO_4_ with a solution of NaNO_3_, H_2_SO_4_, and graphite [19]. It requires a huge quantity of concentrated acid and KMnO_4_ to guarantee enough oxidation. Furthermore, the previously mentioned approaches cause severe environmental contamination and metallic impurities on the GO layers. In particular, about a thousand-fold more water than graphite is required in the Hummers’ method to get rid of the excess of H_2_SO_4_ and KMnO_4_ subsequent to the oxidation reaction, which leads to an enormous amount of sewage water comprising heavy metal ions and strong acids. Furthermore, they are particularly time-consuming, since they may require more than 100 h for oxidation. Many modifications of these approaches, especially of the Hummer’s one, have been published, with enhancements being constantly investigated to attain improved results at a lower cost. For example, strategies without NaNO_3_ in order to avoid the formation of toxic gasses (NO_2_/N_2_O_4_) and to make the disposal of waste water easier due to the absence of Na^+^ and NO_3_^−^ ions have been reported [20]. GO can also be prepared from graphite oxide by means of sonication, stirring, or even by rapid freezing of an aqueous solution containing the parent oxide, which is followed by thawing of the resulting solid [21]. Sonication is a time-effective technique for completely exfoliating graphite oxide, while it can seriously impair the graphene flakes, which decreases their dimensions from μm to nm, and even leads to graphene platelets. Mechanically stirring is not such a clumsy approach, even though it involves longer periods.

Recently, electrochemical processes have been employed to synthesize carbon nanomaterials due to of their environmentally-friendliness, high efficiency, and inexpensiveness [22]. A reduced graphene with a very high C/O ratio (25.3) and high electron mobility has been synthesized within seconds by electrochemical exfoliation of graphite foil [23]. Analogously, GO has been produced by electrochemical oxidation of several graphitic materials in a variety of geometries such as pencil cores and graphite powders, foils, rods, or plates [24,25,26,27]. The reported approaches have emerged as promising and versatile alternatives to the chemical methods. By controlling the processing parameters, such as applied potentials and currents, exfoliation time, as well as electrolyte composition and temperature, GOs with different defect densities, oxygen content, graphene layer numbers, and lateral sizes have been obtained [27]. Moreover, chemical reactions with functionalizing agents can occur during the electrochemical exfoliation to produce in situ chemically functionalized (doped) GO-based materials [28]. However, the electrolysis process generally worsens the expansion and delamination of the graphitic materials, which leads to products with low oxidation and exfoliation levels, with properties that considerably differ from the GOs prepared by the conventional methods mentioned previously. Furthermore, electrochemical exfoliation methods have demonstrated poor controllability in the structural and physical properties of the resulting graphene materials, and typically lead to low-yield production [29]. Overall, despite noteworthy research efforts over recent years focusing on GO fabrication by electrochemical exfoliation from graphite, very few studies have succeeded. Therefore, further investigation in this direction is required to develop scalable, safe, rapid, and green methods. In this regard, there are two critical challenges that should be addressed. First, there is still no simple electrochemical exfoliation process that can precisely control the chemical composition and structure of the resulting graphene materials. The approaches recently reported typically lead to heterogeneous mixtures containing traces from the raw materials such as partially oxidized and fully oxidized flakes, and comprise layers with different defect content, lateral size, and number of functional groups. Second, the methods also face a compromise between the yield and the property control of the resulting materials. The development of cost-effective electrochemical exfoliation means to fabricate GOs by targeting specific applications remains an open question.

In this context, for the first time, we have developed a straightforward, green, and inexpensive electrochemical method that enables users to finely control the level of GO oxidation and exfoliation by carefully modifying the synthesis conditions, which is of large interest from an application standpoint. A wide range of experimental conditions have been tested to optimize the synthesis process in order to obtain homogeneous GOs that preserve the integrity of the graphene flakes, without remnants of the raw material and at a good yield. The aim is to maximize their oxidation and exfoliation levels, which, consequently, would improve their solubility in aqueous solutions and their aptitude to interact with hydrophilic and amphiphilic molecules such as proteins, phospholipids, DNA, and more. The idea of the method is to split the GO preparation process into two stages: a mild intercalation stage of SO_4_^2−^ ions within the graphite layers leading to a graphite intercalation compound (GIC) and then an oxidation/exfoliation stage of the GIC under stronger conditions. Thus, the voltage and time of both stages as well the concentration of the electrolyte in the exfoliation step varied in order to assess their effect on the degree of exfoliation, defect content, and level of oxidation of the resulting GOs. An unprecedented minimum C/O value was obtained for the optimal conditions. The developed method provides better yields than a reference GO synthesized from graphite via a modified Hummers’ method.

## 2. Materials and Methods

### 2.1. Materials and Reagents

High purity flexible graphite foil (FGF, d_25 °C_ = 1.00 g/cm^3^, C: 99.5%, S <300 ppm, Cl < 50 ppm, ash < 1%, thickness 0.1 mm) was provided by Beyond Materials, Inc. (Tucson, AZ, USA) and dried in an oven at 60 °C for 48 h before use. Powdered graphite flakes (SP-1, d_25_ °C = 1.05 g/cm^3^, C: 99.9%, ash < 0.5%, average size 30–150 μm) were acquired from Bay Carbon, Inc. (Michigan, MI, USA) and subjected to identical treatment. KMnO_4_, H_2_SO_4_, K_2_S_2_O_8_, P_2_O_5_, H_2_O_2_ (30 wt % in water), and platinum wire (ø: 0.5 mm, 99.99% trace metals basis) were obtained from Sigma-Aldrich (Madrid, Spain) and used as received. Ultrapure water was purified by a Millipore Elix 15,824 Advantage 15 UV system (Millipore, Milford, MA, USA).

### 2.2. Synthesis of Electrochemically Exfoliated Graphene Oxides (EGOs)

The EGOs were synthesized from FGF via an electrochemical process at room temperature (25 ± 2 °C) that comprised two stages. The first stage was carried out in an electrolysis cell, which had a slice of FGF stuck onto a tungsten wire through silver glue as an anode, a Pt wire as a cathode, and 98 wt% H_2_SO_4_ diluted in 100 mL of Milli-Q water as an electrolyte. A static voltage of 1 or 2 V was initially applied for 10 or 30 min, which led to formation of a turquoise-blue graphite intercalation compound (GIC). This low voltage aided to wet the carbon material and induced the mild intercalation of SO_4_^2−^ ions within the interlayers of FGF [30].

**Figure 1:**
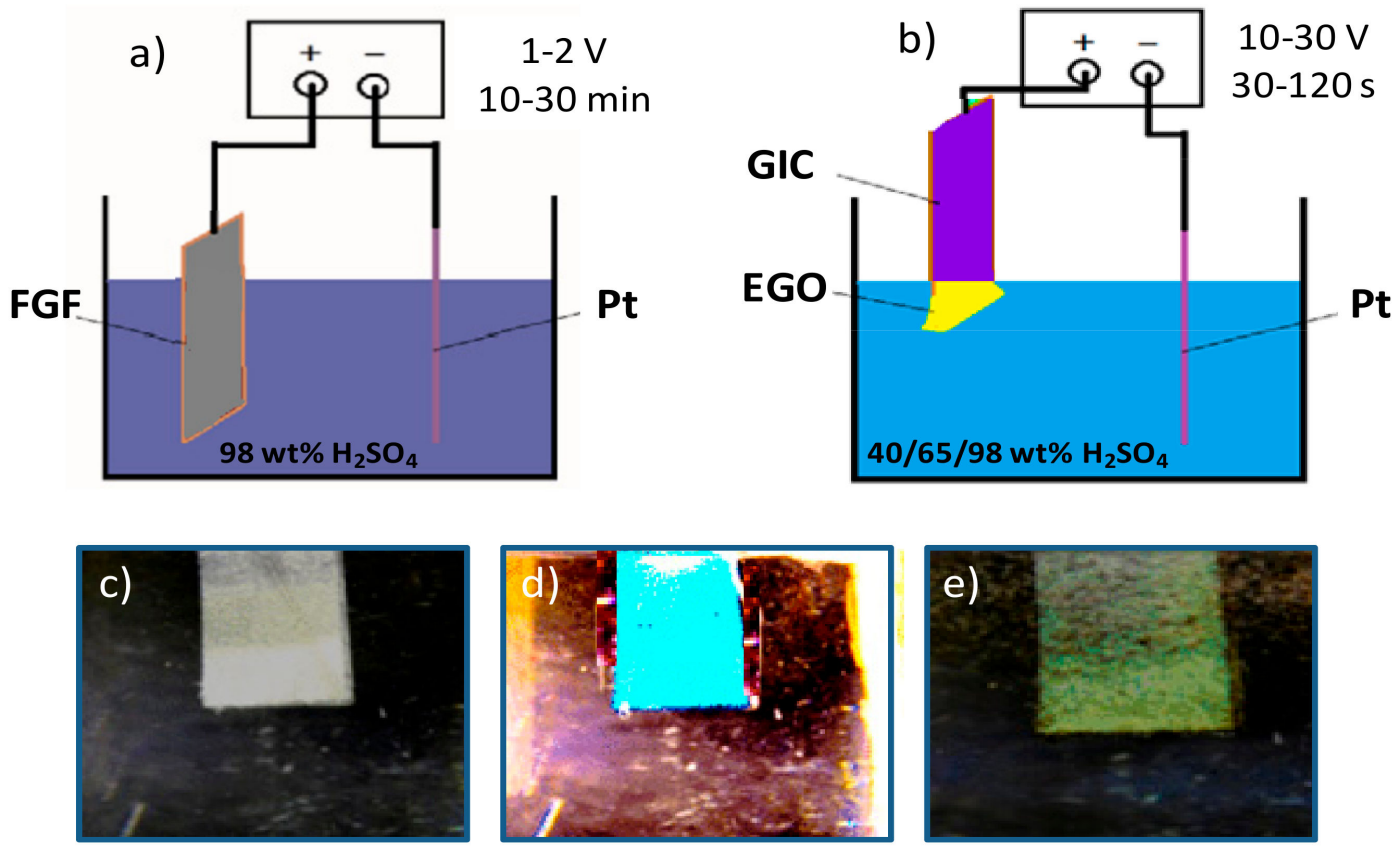
Top: Schematic representation of the electrolytic cells used for the synthesis of GO: (**a**) intercalation step, and (**b**) oxidation/exfoliation step. Bottom: Images of grey FGP (**c**), turquoise-blue GIC, (**d**) and yellowish synthesized EGO (**e**).

Prior to the second step, the synthesized GIC was drawn out and pressed to eliminate the absorbed H_2_SO_4_. Then, the electrochemical oxidation was performed using the GIC as an anode, a Pt wire as a cathode, and 40, 65, or 98 wt% H_2_SO_4_ diluted in 100 mL of Milli-Q water as an electrolyte. A high voltage ranging from 10 to 30 V was applied for different periods between 30 and 120 s. The high bias caused the electrolytic oxidation of the GIC, which results in the formation of a yellowish oxidized graphite oxide that was readily exfoliated into graphene oxide during the voltage application. A schematic representation of the experimental setup used for the electrochemical synthesis of the EGOs and typical images of FGF, GIC, and EGO are shown in Figure 1.

The exfoliated graphene oxide sheets were collected by filtration, cleaned with water, purified via centrifugation at 2500 rpm to remove undesirable large particles, and further exfoliated in water by ultrasonication for 30 min at a power of 140 W, which leads to well dispersed EGO. After vacuum-freezing drying, the yields of the synthesized EGOs were calculated to range from 121% to 137%. The experimental conditions, namely bias voltage and time of the intercalation and exfoliation stages, as well as concentration of the electrolyte in the exfoliation stage varied in order to determine their effect on the degree of exfoliation, defect content, and level of oxidation of the resulting EGO products. The different exfoliation conditions tested along with the nomenclature of the EGO samples obtained herein are summarized in Table 1. For comparative purposes, a GO was also synthesized from flake graphite via a modified Hummers’ method, as reported elsewhere [31], in the presence of a mixture of concentrated K_2_S_2_O_8_, H_2_SO_4_, and P_2_O_5_ at 80 °C for a few hours. The product was filtered, dried, and oxidized for a second time by adding H_2_SO_4_, KMnO_4_, and cold water. Excess of KMnO_4_ was decomposed through a subsequent addition of 30 wt% H_2_O_2_ and 5 wt% HCl aqueous solutions. Afterward, the product was filtered anew, purified, bath sonicated for half an hour at 150 W, and vacuum freeze-dried. The calculated reaction yield was 112%.

### 2.3. Characterization

A LECO CHNS-932 elemental analyzer was used to perform elemental analysis measurements. Chromatograms were acquired with a 7820A Gas Chromatograph coupled with a 5975 mass spectrometry system equipped with a 30 m × 0.25 mm × 0.25 μm HP-5MS capillary column. The injector and detector temperature were 250 and 150 °C, respectively, and the oven temperature program was a ramp from 50 to 100 °C for 10 min, which is followed by another ramp until 230 °C for 15 min. The carbon/oxygen atomic ratio (C/O) was calculated according to the method described by Kaspar [32].

A SU8000 Hitachi scanning electron microscope operating at 15.0 kV and an emission current of 10 mA was employed to acquire the scanning electron microscopy (SEM) images. The microscope is fitted with an energy-dispersive X-ray (EDX) detector that allows for qualitative and quantitative analysis of the sample composition.

A Philips Tecnai 20 FEG electron microscope fitted with a LaB6 filament, working at 200 kV, was used to obtain the transmission electron microscopy (TEM) micrographs with point-to-point resolution of 0.3 nm.

Atomic force microscopy (AFM) imaging was performed using a Bruker Dimension Icon system coupled with a Nanoscope V controller, using a Peakforce QNM imaging mode and a 100 μm long monolithic silicon cantilever.

A Bruker D8 Advance diffractometer fitted with a Cu X-ray tube and a Ni K_β_ filter was used to carry out the X-ray diffraction (XRD) measurements, working at 40 kV and at an intensity of 40 mA.

Mid-range room temperature Fourier-transformed infrared (FT-IR) spectra were collected with a Perkin Elmer Frontier FTIR spectrophotometer fitted with an attenuated total reflectance (ATR) sampling unit and a diamond window. Thirty-two scans were recorded for each sample with 1 mW laser output and a resolution ≥4 cm^−1^. Previous to the measurements, the nanomaterials were milled and mixed with KBr, and then pressed into a pellet. Atmospheric compensation was carried out.

Raman spectra were acquired at room temperature with a Renishaw Raman microscope set with a He-Ne gas laser with a wavelength of 632.8 nm and an output of 1.0 mW. To improve the signal-to-noise ratio, at least 10 scans were recorded for each sample.

The room temperature electrical resistivity of the synthesized EGOs was determined under a pressure of 600 kPa set by using an upper weight, with a KEITHLEY 2182A nanovoltmeter and a KEITHLEY 6221 current source, respectively. Prior to the measurements, each sample was positioned in a Teflon cylinder and compressed for 1 h between two stainless steel plates that acted as electrodes.

## 3. Results and Discussion

### 3.1. Oxidation Level of the Synthesized EGOs

Elemental analysis was used to determine the absolute amounts of C, H, N, and S elements of the synthesized EGOs [33], and to calculate their carbon/oxygen (C/O) atomic ratio, which is indicative of the degree of oxidation. Therefore, this shows the effectiveness of the electrochemical oxidation process of graphite. Typical C/O atomic ratios for GO lie between 2.1 to 2.9 [34]. The reference GO synthesized by the Hummer’s method shows a C/O ratio of 2.25, which is in good agreement with previous studies [7]. The EGOs display a wide range of C/O ratios (1.46–2.96, Table 1), which indicates that some of them present higher and other lower degrees of oxidation than the reference, which corroborates the strong influence of the synthesis conditions on the level of oxidation of the final sample. In particular, when the voltage of the second stage is higher than 20 V, the exfoliation rate should be very fast, which results in large GO aggregates and thick flakes (i.e., ≥20 nm for EGO 21 and 22, as corroborated by TEM, Section 3.2). Likely, only the lateral parts of the sheets are oxidized, which results in lower oxidation degrees. A similar result is obtained when the low voltage is applied for a long time (i.e., 30 min): the FGF sheets are easily broken into small pieces during the intercalation stage and are poorly oxidized, which leads to high C/O ratios. Furthermore, too much swelling of the GIC can occur if the intercalation time is too long, which leads to a strong decrease in mechanical strength and conductivity, and, therefore, it is more difficult to be further oxidized. In contrast, syntheses performed using concentrated H_2_SO_4_ during the second stage, result, as expected, in a very high level of oxidation. Thus, an unprecedented minimum C/O ratio value of 1.46 has been obtained when a low bias of 2 V was applied for 10 min and the oxidation stage was performed under 20 V for 60 s using 98% H_2_SO_4_ as an electrolyte. This particularly highly oxidized GO should have a very high content of carboxylic acid groups, which will be discussed in a following section, preferentially located at the sheet edges, which are more easily oxidized. Nonetheless, due to its elevated oxygen content, besides the conventional epoxy/hydroxyl groups situated on the basal planes, it could also comprise other oxygen-containing functional groups such as ketone/quinone (C=O), carboxylates (O–C=O), and even peroxy groups (O–O) [35]. This sample (EGO 15) also exhibits a very high level of exfoliation according to TEM analysis. When the low voltage was decreased to 1 V, and the rest of the conditions were maintained, the C/O ratio increased to 1.54, indicating that the voltage of the first stage is also crucial to attain a good wetting of the sample and allow the intercalation of ions within the graphite sheets, thus promoting their exfoliation. Conversely, when the same conditions were applied, albeit the concentration of the electrolyte in the second stage, was reduced to 65% or 40%, the C/O ratios increased up to 1.79 and 1.91, (EGO 5 and 4, respectively), indicating a less efficient exfoliation and oxidation, as also corroborated by TEM analysis.

Very good results (C/O = 1.67) have also been attained when a low bias of 2 V was applied for 10 min and the oxidation stage was performed under 20 V for 60 s using 65% H_2_SO_4_ as an electrolyte (EGO 14). However, when the 20 V were only applied for 30 s, the C/O ratio noticeably increased, which indicates partial oxidation of the sheets. However, when the same voltage was applied for 120 s, the same C/O ratio was attained (1.69). This suggests that there is an optimum time for the oxidation reaction, close to 60 s, and longer oxidation periods do not lead to higher oxidation degrees because a saturation level in the number of functional groups located onto the basal planes and at the edges has been attained. This is consistent with previous theoretical studies on the distribution of oxygenated groups onto GO sheets, which reveal that epoxide and hydroxyl moieties prefer the island arrangement rather than a homogeneous distribution [36]. In the island configuration, the functional groups are very close to each other, which makes it difficult to incorporate more groups and leads to a saturation level that has been calculated to be around 9 and 8 epoxide and hydroxyl units, respectively [36]. This is also in agreement with the observations made during the electrochemical syntheses: for a given set of conditions, with increasing exfoliation time from 30 to 60 s, the yellowish areas of the EGOs increased, and, in general, the whole surface became yellow at around 60 s. However, no color changes were observed after 1 min. Only when mild conditions were applied (a low bias of 1 V for 10 min followed by 10 V for 60 s and 65% H_2_SO_4_ as an electrolyte), a slight decrease in the C/O ratio (~5.3%) was found when increasing the exfoliation time from 60 to 120 s.

On the other hand, it is important to highlight the very good agreement found between the C/O ratio. Hence, the percentage of C and O was detected by elemental analysis and EDX (Table 1), despite the fact that EDX is a surface technique while elemental analysis gives the value of the whole sample. Thus, the good consistency between the results obtained by the two techniques confirms that the samples are homogeneous over the entire volume. It should be noted that the C/O ratio influences structural properties like the degree of exfoliation and number of layers, amount of defects, flake size, and concentration of functional groups, among others [37], which, in turn, would tailor the band structure, hence the photoluminiscent behaviour, as well as the electrical and mechanical properties. In addition, the oxygenated moieties provide anchoring points for subsequent chemical modification [38].

### 3.2. Morphological Characterization of the EGOs

**Figure 2:**
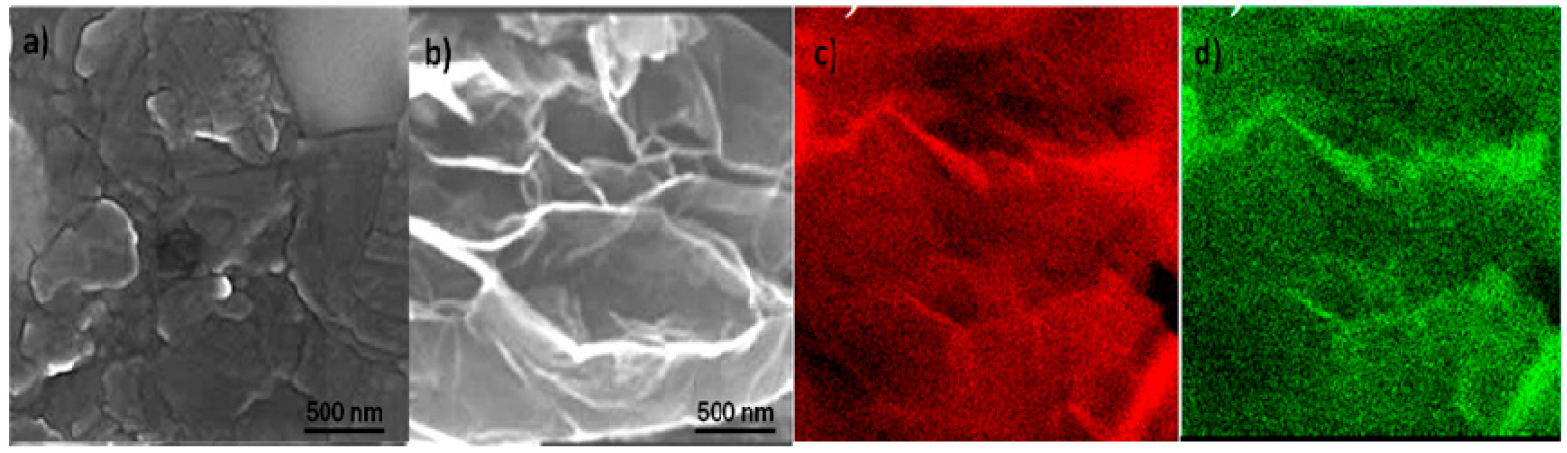
SEM images of pristine FGF (**a**), EGO 14, (**b**) and the EDX maps of the area shown in b for carbon (**c**) and oxygen (**d**).

The surface morphology of FGF and the synthesized EGOs was investigated by SEM, and typical images of FGF and EGO 14 are compared in Figure 2. The image of FGF (Figure 2a) reveals a dense and compact surface structure, composed of large grains with different shapes, and a smooth and flat surface. This fine crystal grain structure is consistent with its crystalline nature. In contrast, the EGO (Figure 2b) shows a rough surface topography with exfoliated and well separated graphene sheets, with thicknesses ranging from 30 to 10 nm. Furthermore, the flakes are highly-wrinkled, which indicates a deformation of the graphene layers due to the linkage of the oxygenated functional groups after the electrochemical process. Furthermore, the grain pattern cannot be observed, which suggests a reduction in the level of crystallinity, while large nanosheet sizes are preserved. Overall, the images corroborate that the electrochemical process used herein maintains the integrity of the graphene flakes, and enables us to obtain large area uniform GO sheets. On the other hand, the EDX maps of EGO 14 are shown in Figure 2c,d, from which the C/O ratio was obtained (Table 1).

**Figure 3:**
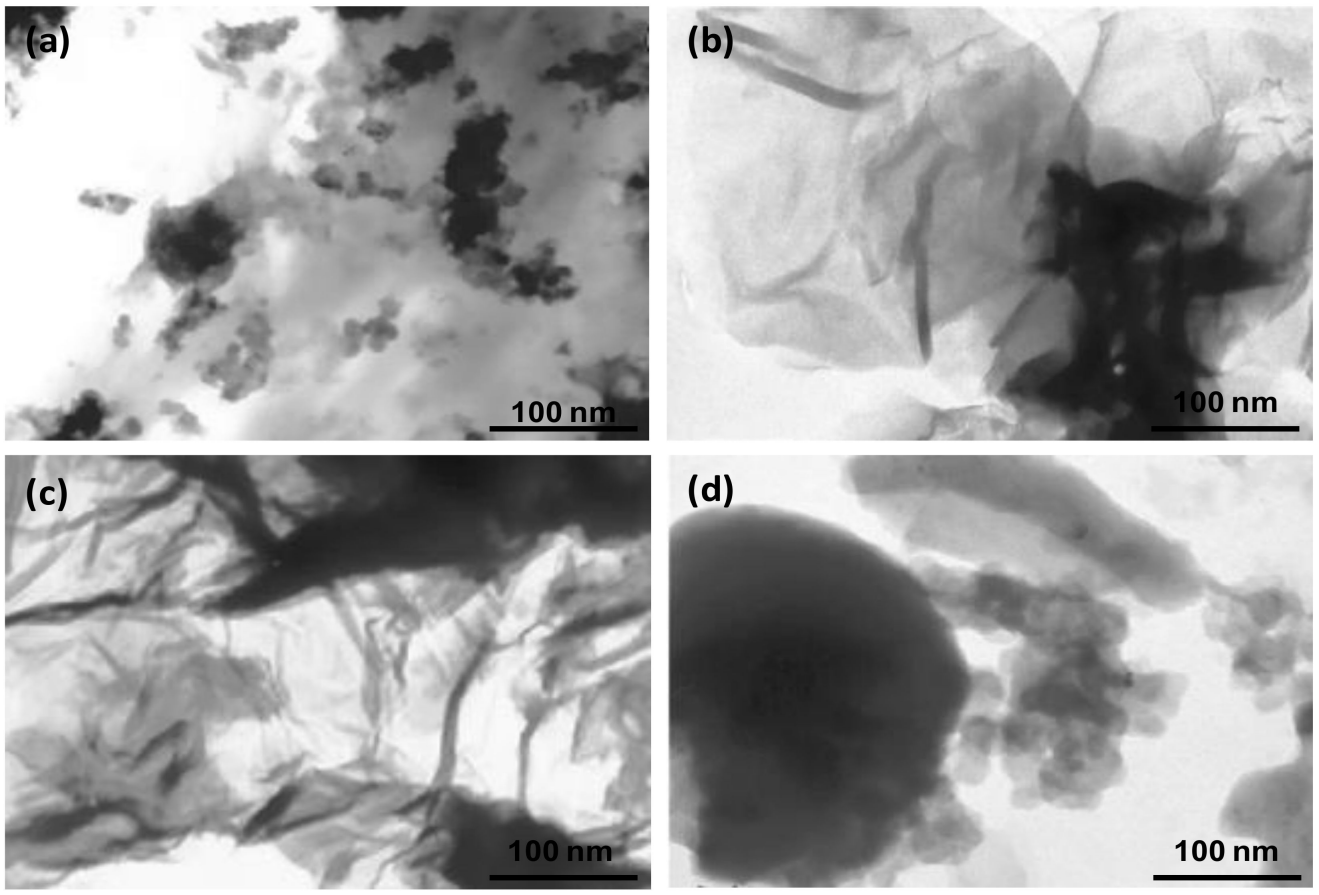
TEM images of EGO 4 (**a**), EGO 14 (**b**) EGO 15 (**c**), and EGO 21 (**d**).

Further information about the surface morphology of the samples was attained by TEM, and typical images of EGO 4, 14, 15, and 21 are compared in Figure 3. A moderate level of exfoliation can be observed for EGO 4 (Figure 3a), which was prepared by applying mild conditions: a low bias of 1V for 10 min and 60 s of exfoliation under 20 V in 40% H_2_SO_4_ as an electrolyte. Although no FGF is found, corroborating that it has been oxidized and exfoliated during the electrochemical process, leads to GO flakes with thicknesses <20 nm. Some small GO aggregates can be observed, which are indicative of a less efficient exfoliation process.

In contrast, TEM images for EGO 14 and 15 (Figure 3b,c) clearly reveal the formation of homogeneous and good quality GO sheets, with a clear flake structure and lack of traces from the raw material. Very well exfoliated, uniform, and thin sheets can be detected, in particular for EGO 15, that exhibits highly wrinkled sheets with thicknesses <5 nm. Such a high degree of bending is consistent with the presence of a large number of oxygenated functional groups that can interact via H-bonding [35]. Furthermore, the darker areas are somehow light transparent, which also suggests a good level of exfoliation. Thus, the application of 20 V for 60 s with concentrated H_2_SO_4_ as an electrolyte is demonstrated to be a very effective exfoliation approach. In the case of EGO 14, prepared under the same conditions despite using 65% H_2_SO_4_ as an electrolyte, the sheets are slightly thicker and less bended. While they appear homogenous, their level of exfoliation is also very good and preserves the integrity of the graphene flakes. Conversely, for EGO 21 (Figure 3d), very large blocks of GO aggregates can be observed, and the darker areas reveal small, partly oxidized GO pieces or even some remaining FGF precursors. Since a high voltage of 30 V was applied during the oxidation stage, the exfoliation rate should be very fast, resulting in large GO aggregates and flakes with thicknesses ≥20 nm. Analogous morphology was found for EGO 22 prepared by applying the same high voltage during a longer time (120 s). Furthermore, during the synthesis of these samples, the low bias was applied for a long period (30 min), which likely results in a very high level of swelling. This results in poorer mechanical strength. Hence, the sheets are easily broken into small pieces difficult to be oxidized. Thus, very similar morphology, albeit with smaller aggregates and slightly thinner sheets was found for EGO 19 and 20, which were also synthesized by initially applying 2 V for 30 min.

AFM measurements were also performed to estimate the average thickness of the EGO flakes, and the results are summarized in Table 1. Theoretically, the thickness of single layer graphene is 0.345 nm. However, it has been reported that GO layers are about 1.1 nm [39]. A wide range of thickness values have been measured for the EGOs, which range between 3.8 and 18.6 nm. This defines them as few-layer GO materials according to referential nomenclature [40]. The trend observed is consistent with that obtained from both TEM and SEM observations. GOs with the highest level of exfoliation (EGO 12 and 15) display the lowest thicknesses. In general, with an increasing C/O ratio, upon decreasing the sample oxidation level, the thickness increases.

The average lateral dimensions of the EGOs were also estimated by AFM. The reference GO has an average lateral dimension of 24 μm. Samples synthesized by applying the low voltage for 30 min (EGO 7, 8, 19–22) show the smallest dimensions in the range of 4–12 μm. The FGF sheets could be broken into smaller pieces during the long intercalation stage, which results in smaller layers. Samples prepared using concentrated H_2_SO_4_ during the second step (EGO 3, 6, 12, 15) have lateral sizes between 10 and 22 μm, while the rest of the nanomaterials show dimensions larger than 20 μm.

### 3.3. FT-IR Analysis of the EGOs

IR spectroscopy was used to characterize the functional groups present onto the synthesized EGOs and get some insight about their oxidation level. GO is known to contain epoxide, hydroxyl, ketone, and even peroxy groups on the basal planes, while carboxyl moieties are located at the borders. Note that, due to the variety of oxygenated groups and the overlapping modes below 1500 cm^−1^, the interpretation of the FT-IR spectra of GO is complex and it is not possible to unambiguously correlate specific frequencies to a particular functional group [41].

**Figure 4:**
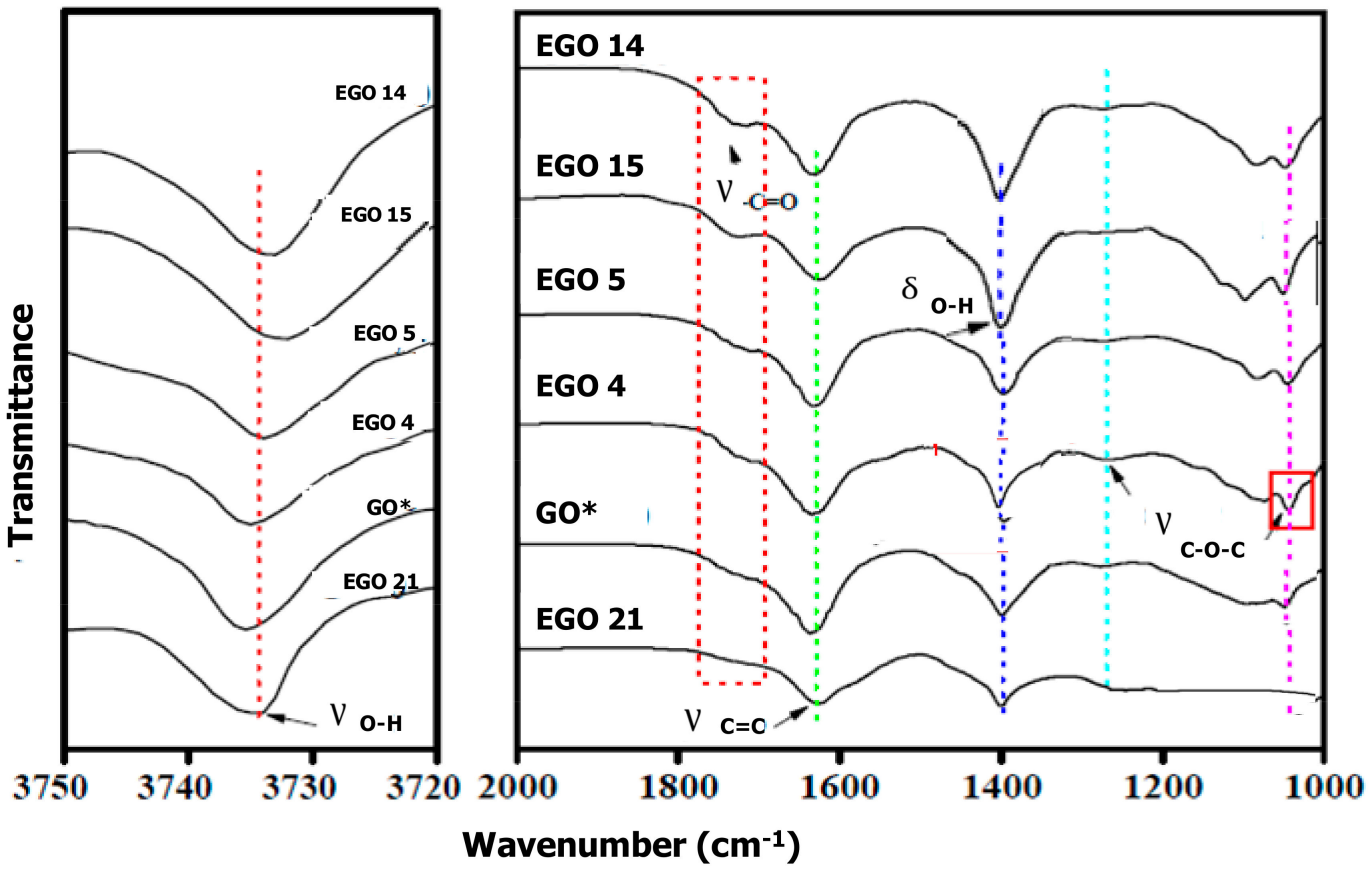
FT-IR spectra of the reference (GO*) and the indicated EGO samples.

The spectrum of the reference GO (Figure 4) shows a broad peak centred at ~3735 cm^−1^ assigned to the O–H stretching vibration, another at ~1740 cm^−1^ characteristic of the C=O stretching of the COOH moieties [42], one at around 1630 cm^−1^ corresponding to the C=C stretching of aromatic rings, the other at about 1400 cm^−1^ that matches with the O–H bending vibration and bands at around 1270, and 1050 cm^−1^ associated with the C–O stretching in epoxy groups [43]. All these features are also present in the EGOs, which suggests that they have the same oxygen-containing functional groups as the reference GO. Nonetheless, noticeable changes in the peak positions and intensities can be observed among the spectra of GO and the different EGOs that point toward diverse levels of oxidation. Thus, the EGO 15 displays the wider and most intense O–H stretching band, which is in agreement with its very low C/O ratio (Table 1). This signifies a very high content of carboxylic acid groups. Furthermore, the band is displaced toward lower wave numbers, which is symptomatic of the presence of strong hydrogen bonds [44]. These are more characteristic among carboxylic acid groups. The EGO 14 also exhibits an intense band shifted toward lower frequency, while, in the reference GO, the band appears at slightly higher wave numbers, which suggests weaker/fewer H-bonds.

A clearer trend can be observed from the O–H deformation band, which suggests the following level of oxidation: EGO 21 < GO* < EGO 4 < EGO 5 < EGO 14 < EGO 15. These results are perfectly consistent with those derived from EDX and elemental analyses. Thus, the EGO 21 prepared by applying a very high voltage during the second stage shows the lowest level of oxidation, which is in agreement with the presence of large aggregates and thick flakes as revealed by TEM. Analogous spectrum, even with a weaker band, was obtained for EGO 22, prepared under similar conditions despite applying the voltage of the second stage for a longer time and using 65% H_2_SO_4_ as an electrolyte. Conversely, EGO 14 and 15, prepared by applying a low bias of 2 V for 10 min, which is followed by 20 V for 60 s using 65% or 98% H_2_SO_4_ as an electrolyte, respectively, display the most intense band, which suggests that these are optimal conditions to attain a very high level of flake oxidation. On the other hand, EGO 4 and 5, prepared under mild conditions, display moderately intense bands, suggesting intermediate levels of oxidation. Nonetheless, the band intensity is stronger for EGO 5 compared to the other one, which corroborates that the concentration of the electrolyte strongly influences the number of oxygen-containing groups formed during the second stage.

Regarding the band at ~1740 cm^−1^ related to the C=O stretching of the COOH groups, it is clearly more intense for EGO 14 and 15, while it can hardly be detected in EGO 21 and it shows very low intensity in the reference GO. A similar trend is found for the peaks assigned to epoxy groups, which cannot be found in EGO 21, while are very intense in EGO 15. Furthermore, this sample displays a new peak at about 1135 cm^−1^ likely ascribed to ether groups that cannot be found in the other EGOs, which is consistent with its high level of oxidation [45].

### 3.4. Raman Analysis of the EGOs

**Figure 5:**
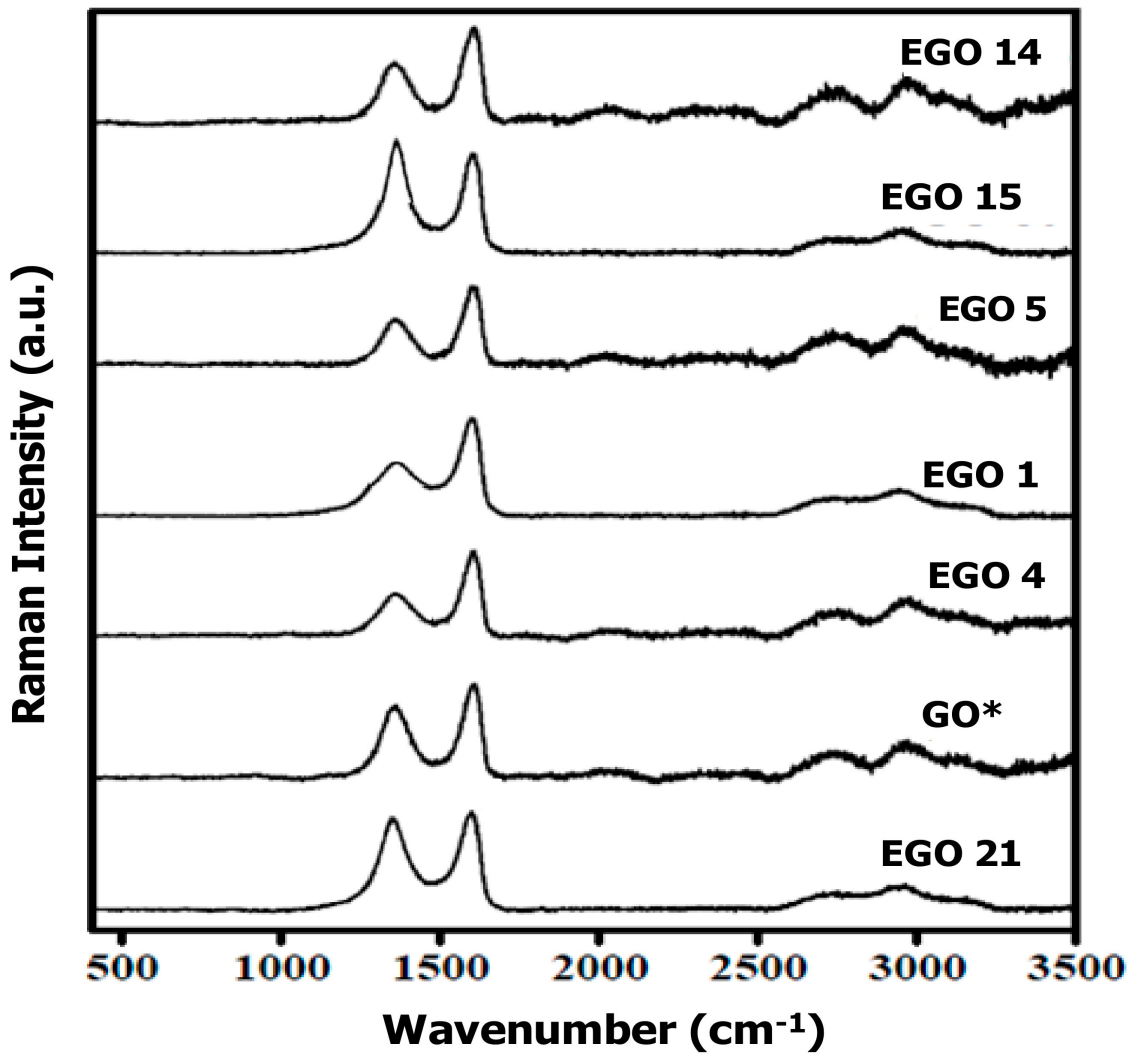
Raman spectra of the reference (GO*) and the indicated EGO samples.

Raman spectroscopy is a useful tool to assess the number of defects in carbon nanomaterials as well as their crystallite size and number of layers [46]. In particular, it is a widely used technique to characterize GOs with different oxidation degrees [47]. The Raman spectrum of GO shows general features at about 1360, 1600, and 2700 cm^−1^ that correlate with the D, G, and 2D peaks, respectively. The G band corresponds to the E2g vibrational mode found in a graphite single crystal [48], which is characteristic of sp^2^ hybridization. The D band is associated to defects, vacancies, or lattice disorders due to the binding of oxygen-functional groups and the 2D band is attributed to second order phonon processes [49]. These features are observed in the spectra of all the EGO samples with differences in the peak positions and their intensities (Figure 5). Thus, the I_D_/I_G_ ratio (the integrated intensity ratio of the D peak and the G peak) is an indication of the quality of the GO sheets and their number of defects [50]. One of the typical defects is the double vacancy (C2), also known as 5-8-5 defect, which comprises one octagonal and two pentagonal rings [51]. In addition, the common 5-7-7-5 rings, also known as Stone-Wales (SW) defects, are a key factor in graphitic-based materials.

I_D_/I_G_ data ranging from 1.71 to 0.41 have been calculated for the synthesized EGOs (Table 1). Clearly, EGO 19, prepared under strong oxidizing conditions (2 bias for 30 s followed by 10 V for 30 s and 98% H_2_SO_4_ during the second stage) shows the highest I_D_/I_G_ value, which is followed by EGO 15, and is synthesized with a shorter swelling stage despite a longer oxidation step in 98% H_2_SO_4._ This sample also displays a small shift of the G band toward higher frequency (blue-shift), which is indicative of a high disorder [48]. A high I_D_/I_G_ ratio was systematically obtained for all the EGOs prepared using 98% H_2_SO_4_ as an electrolyte, which is symptomatic of GO sheets with a large amount of surface defects generated in the presence of the concentrated acid. An elevated I_D_/I_G_ value, higher than that of the reference GO, was also obtained for EGO 21 and 22, which was prepared by applying the low bias for a long period (30 min). This is likely related to their high level of swelling, which results in poorer mechanical properties. Hence, the sheets are more easily damaged and generate a larger amount of defects.

Among the 22 EGOs synthesized, 14 show lower I_D_/I_G_ than the reference, which indicates a superior quality of their GO flakes_._ For instance, an intermediate I_D_/I_G_ value of 0.87 was attained for EGO 14. Although this sample exhibits a high level of oxidation and a good degree of exfoliation, as corroborated by TEM, its amount of surface defects is somewhat low, likely because 65% H_2_SO_4_ was used as an electrolyte. Lower ratios were obtained for EGO 4 and EGO 5, prepared by applying milder conditions, despite their oxidation level was also poorer. Moreover, EGO 1 prepared under the softest conditions, shows the lowest I_D_/I_G_ value.

On the other hand, I_D_/I_G_ has been widely used to estimate the in-plane crystallite size (L_a_) in non-ordered nanostructures [48]: L_a_ = 2.4 × 10^−10^ λ^4^ (I_D_/I_G_)^−1^. Thus, the larger the I_D_/I_G_ ratio, the smaller L_a_, that is, the crystalline regions would decrease in size as the structural disorder increases. L_a_ values ranging from 2.8 to 13.4 nm have been calculated for the synthesized EGO samples, which indicates a strong effect of the synthesis conditions on the crystallite size. This is in good agreement with recent works [52].

### 3.5. X-ray Diffraction Study of the EGOs

**Figure 6:**
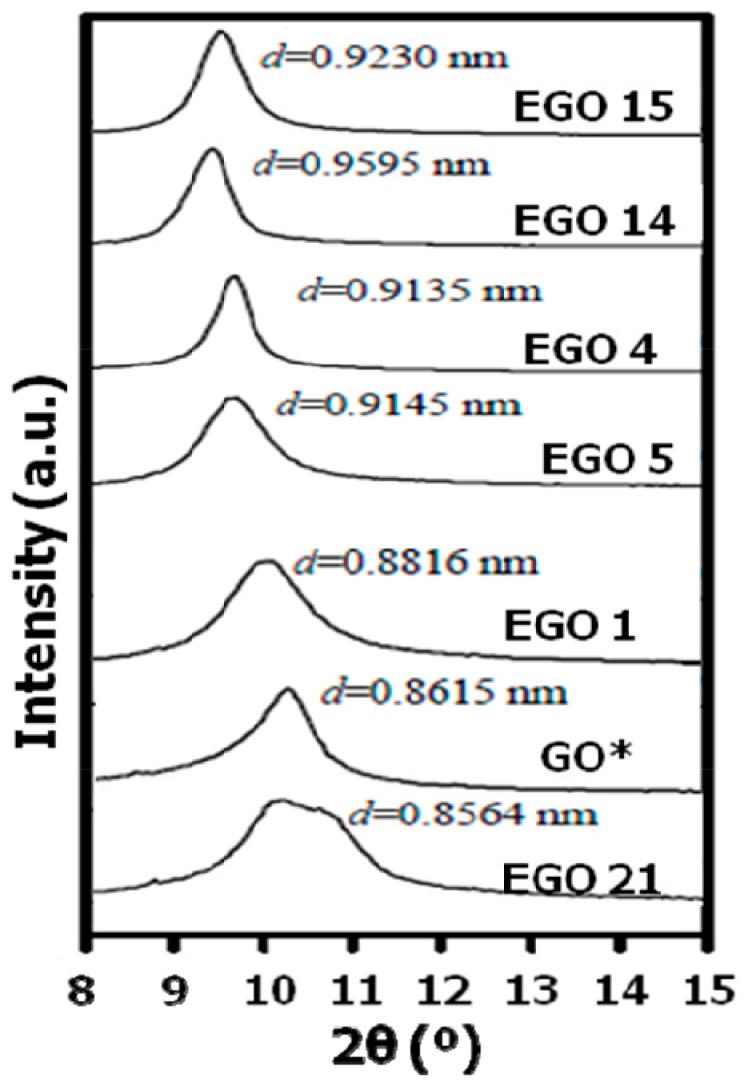
X-ray diffractogram of the reference (GO*) and the indicated EGO samples.

X-ray diffractograms were acquired to investigate possible changes in the interlayer spacing (*d*) and crystallite size depending on the synthesis conditions of the EGOs (Figure 6). As known, raw graphite shows an intense diffraction peak at 2θ = 26.5°, which corresponds to a *d* value of 0.336 nm taking into account the Bragg’s equation. This peak was not observed in any of the synthesized EGOs, since it disappeared after oxidation, while a new peak appeared in the range of 9–12° corresponding to the (001) reflection of GO [53]. The presence of this characteristic peak corroborates that all the samples were partially or completely oxidized during the electrochemical process.

The *d*-spacing calculated for the reference GO is 0.8615 nm, about 2.6-fold bigger than the calculated for graphite. This increase in the *d*-spacing is consistent with previous works [37], which is accredited to the presence of oxygen containing groups, mainly epoxide and hydroxyl formed during the oxidation process, laying between the GO sheets, and a change in the hybridization state of the C atoms from sp^2^ to sp^3^ [54]. The values of *d*-spacing calculated for all the EGOs are listed in Table 1. EGO 19 shows the smallest *d* value, likely due to its low oxidation level and the fact that the oxidation stage was carried out in strong H_2_SO_4_. Hence, most of the oxidation likely occurred on the flake edges. Consequently, the number of oxygen-containing groups within layers is relatively small. This explanation could also account for the fact that EGO 14 presents a lager d value that EGO 15, despite the lager oxygen content of the latter sample calculated by elemental analysis and EDX (Table 1). Thus, systematically, all the samples synthesized using strong H_2_SO_4_ as an electrolyte display a smaller *d* value than expected, according to their oxygen content. The largest interlayer spacing, close to 0.96, was obtained for EGO 14, corroborating that the sample was almost completely oxidized. Longer exfoliation times resulted in slightly lower interlayer spacing (EGO 17), likely because it led to more functional groups on the flake edges. On the other hand, EGO 4 and 5, prepared under mild conditions, present intermediate *d* values, which are in agreement with their moderate level of oxidation, despite being higher than that of the reference GO.

The crystallite size was also estimated from the full width at half maximum (FWHM) of the diffraction peak corresponding to the (001) plane, according to the Debye-Scherrer equation [55]. The values obtained ranged from 4 to 12 nm, which were in good agreement with those determined from the Raman spectra.

### 3.6. Electrical Resistance of the EGOs

It is well known that there is a strong relationship between the structure of GO and its electrical conductivity. The performance of GO in electronic devices is based on its electrical properties, which are significantly influenced by the synthesis process, hence on the level of oxidation [56]. GO is reported to be electrically insulating due to the sp^3^ bonds, the elevated density of oxygenated groups, which are electronegative, and the presence of defects and vacancies that lead to a gap in the electronic density of states [57]. The largest contribution to the electrical resistivity comes from the contact resistance between GO particles, which likely will have two sources: tunneling and constriction [58]. Tunneling resistance occurs in GO since it can be depicted as a conductive nucleus delimited by non-conductive regions. In contrast, constriction resistance is attributed to the narrowness of the conducting pathway due to the tiny contact region between particles. This effect would likely be less important due to the elevated aspect ratio of the GO sheets, which will tend to locate parallel to each other after the pressure is applied.

**Figure 7:**
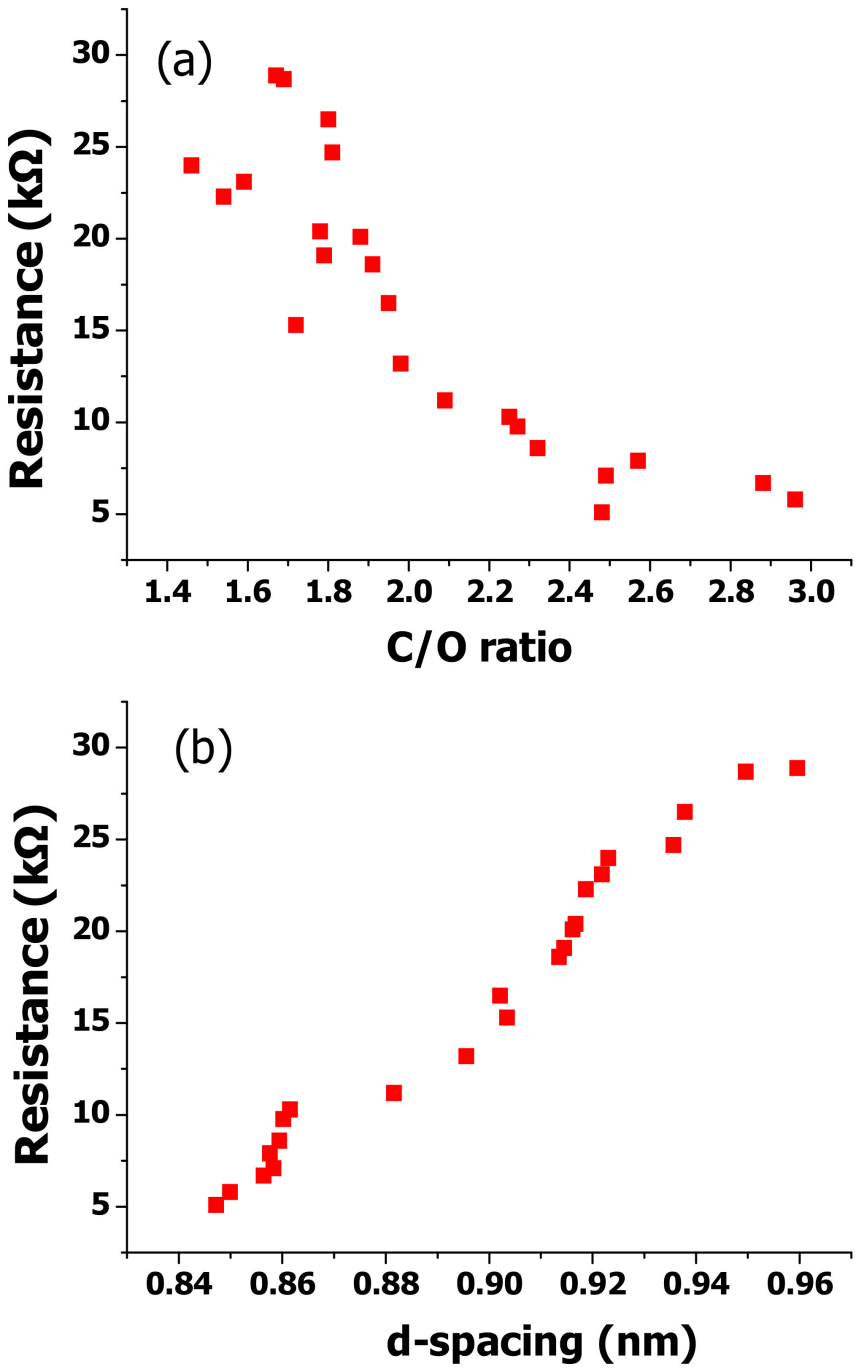
Electrical resistance of the synthesized EGOs versus the C/O ratio (**a**) and the d-spacing (**b**).

Figure 7 compares the electrical resistance of the synthesized EGOs versus their C/O ratio, which is their oxidation level, and their interlayer spacing. It can be observed that the electrical resistance decreases from about 29 to 5 kΩ while the C/O ratio increases (Figure 7a). This is indicative of a trend between the two parameters. Despite not being linear, the electrical conductivity is restored while decreasing the oxidation level. However, EGO 15, which was prepared under strong oxidizing conditions and shows the lowest C/O ratio (1.46) and the second highest I_D_/I_G_ ratio, hence, a very large number of defects, does not exhibit the utmost resistance. Conversely, EGO 14 with a lower oxidation level and I_D_/I_G_ ratio shows the highest resistance value. This suggests that, besides the oxidation level, other factors should govern the conduction mechanism in GO. Theoretical simulations have shown that the conductivities of few-layer graphene nanosheets are reduced when thickness is increased [59]. However, our results do not show a direct correlation between the electrical conductivity and the flake thickness measured by AFM (Table 1).

To analyze the influence of other factors, the electrical resistance was plotted against the interlayer spacing (*d*) calculated from X-ray diffractograms. There is a very clear relationship between both magnitudes: the electrical resistance increases steadily while increasing the interlayer distance, which indicates that the most important factor influencing the conductivity in GO is the tunneling resistance between adjacent sheets. This is consistent with earlier investigations that studied the tunneling effect in carbon nanomaterials [60]. In these materials, the tunneling resistance can be estimated according to the Simmons’s formula [61], which predicts the resistance between two flat electrodes set apart by a small insulating film. Thus, the tunneling resistance increases linearly with a growth in the insulating layer thickness [62]. This is in agreement with the augment found herein with increasing interlayer spacing.

The conduction mechanisms that take place in GO sheets are not well understood yet. Frequent charge will percolate by a hopping conduction mechanism [57], which consists of the transport of charges via localized states. The percolation in random continua or the 2D Swiss-cheese model is a simple way to depict GO conductivity [63]. In this model, the localized states are arbitrarily distributed within the GO material. The model is complicated since the occupation of the localized states is governed by the confluence of the threshold voltage and the local electric field originated by the number of inserted carriers. Hopping between localized states occurs via thermal agitation.

Experimental data reveal a certain dependence of the electrical resistance on the oxidation level. This is consistent with theoretical statistics revealing that the presence of oxygen groups influences the electronic density of states [57]. Thus, the epoxy moieties appearing in all the EGOs can displace the Fermi energy, which significantly influences the electrical resistance. Furthermore, the formation of hydrogen bonds between epoxy and hydroxyl groups could lessen the energy density close to the Fermi level, and consequently, will rise the work function of the carbon nanomaterial [57]. Previous experimental works reported that the work function of GO steadily augments when the C/O ratio increases. This resembles that of graphite for C/O higher than 3 [64]. However, our results suggest that the conductivity is likely conditioned by the type of oxygenated functional groups and their location on the GO flakes.

Therefore, despite that the majority of the models support that the electrical properties of GO are influenced by the number of oxygenated groups, hence, the oxidation level, it is clear from our experimental data that the charge transference depends mostly on the tunneling resistance between neighbouring sheets, hence on the interlayer spacing.

## 4. Conclusions

GO powders with a diverse amount of oxygenated surface groups have been synthesized from flexible graphite foil by an electrochemical two-step process: intercalation and oxidation/exfoliation. A wide range of synthesis conditions have been studied to optimize the oxidation level, by modifying the voltage and time of both stages as well the electrolyte concentration. For comparative purposes, a reference GO was prepared via a modified Hummers’ method. The resulting GO samples have been characterized by different techniques including elemental analysis, SEM, TEM, AFM, XRD, EDX, FT-IR, and Raman spectroscopies as well as electrical resistance measurements. Elemental analysis data confirm the strong influence of the synthesis parameters on the oxidation level. An unprecedented minimum C/O ratio of 1.46 has been obtained by applying 2 V for 10 min followed by 20 V for 60 s using 98% H_2_SO_4_ as an electrolyte. SEM and TEM micrographs reveal the formation of good quality, large, homogeneous and well exfoliated GO sheets. FT-IR corroborates the formation of –OH, C-O-C, C=O, and –COOH surface moieties and points toward strong intermolecular interactions between adjacent groups. A high I_D_/I_G_ ratio in the Raman spectra has been obtained for all the samples prepared using 98% H_2_SO_4_ as an electrolyte, which is indicative of sheets with a large number of defects. Furthermore, a high I_D_/I_G_ is also found for samples prepared by applying the voltage of the first stage for a long period, which is related to their high level of swelling. The interlayer distance between adjacent sheets estimated from XRD was not directly proportional to the oxygen content. Samples prepared using 98% H_2_SO_4_ show smaller interlayer spacing than expected considering their oxidation level, which signifies that a great part of the oxidation took place on the sheet edges. Electrical resistance results do not show a clear trend with the C/O ratio nor with the flake thickness but with the interlayer spacing, which indicates that the electrical performance depends mostly on the tunneling resistance between neighbouring sheets. Overall, the optimal synthesis conditions to attain a high level of oxidation and a moderate number of defects are 2V in the intercalation stage for 10 min, which was followed by 20 V in the oxidation stage for 60 s under 65% H_2_SO_4_. Nonetheless, this sample displays the highest sheet resistance. Overall, the level of oxidation and exfoliation can be tailored by controlling the synthesis conditions, and should be selected depending on the specific application. The approach developed herein provides an efficient way to tune the physical properties of GO-based materials.

## Figures and Tables

**Table 1:** Nomenclature, experimental conditions of the electrochemical synthesis, and characteristics of the different EGO samples.

Sample	Voltage	Time I	Time II	H_2_SO_4_	C/O	O_EDX_	I_D_/I_G_	*d*_001_	*t*
	I/II (V)	(min)	(s)	(wt%)	Ratio	(%)	Ratio	(nm)	(nm)
GO*	_	_	_	_	2.25	31.00	1.04	0.8615	13.8
EGO 1	1.0/10	10	60	65	2.09	33.12	0.41	0.8816	12.1
EGO 2	1.0/10	10	120	65	1.98	35.41	0.47	0.8956	10.7
EGO 3	1.0/20	10	30	98	1.72	40.12	1.01	0.9034	7.14
EGO 4	1.0/20	10	60	40	1.91	36.11	0.59	0.9135	9.13
EGO 5	1.0/20	10	60	65	1.79	38.33	0.70	0.9145	7.11
EGO 6	1.0/20	10	60	98	1.54	44.80	1.37	0.9187	3.98
EGO 7	1.0/30	30	60	40	2.32	29.76	0.43	0.8594	14.4
EGO 8	1.0/30	30	120	65	2.57	26.85	0.54	0.8576	16.5
EGO 9	2.0/10	10	60	65	1.88	36.69	0.79	0.9161	8.75
EGO 10	2.0/10	10	120	65	1.81	38.40	0.69	0.9356	7.37
EGO 11	2.0/20	10	30	65	1.95	35.38	0.74	0.9021	10.2
EGO 12	2.0/20	10	30	98	1.59	43.39	1.51	0.9218	4.24
EGO 13	2.0/20	10	60	40	1.80	38.33	0.77	0.9378	7.53
EGO 14	2.0/20	10	60	65	1.67	41.31	0.87	0.9595	5.84
EGO 15	2.0/20	10	60	98	1.46	47.26	1.68	0.9230	3.79
EGO 16	2.0/20	10	120	40	1.78	38.77	0.93	0.9167	7.26
EGO 17	2.0/20	10	120	65	1.69	40.82	0.99	0.9496	5.92
EGO 18	2.0/30	10	30	65	2.49	27.71	1.22	0.8583	15.8
EGO 19	2.0/10	30	30	98	2.48	27.82	1.71	0.8472	16.0
EGO 20	2.0/20	30	120	65	2.27	30.39	1.23	0.8602	14.5
EGO 21	2.0/30	30	60	40	2.88	23.96	1.30	0.8564	18.6
EGO 22	2.0/30	30	120	65	2.96	23.31	1.39	0.8499	17.9

* Synthesized by a modified Hummers’ method. I and II refer to the intercalation and exfoliation stages, respectively. The C/O ratio has been calculated from elemental analysis measurements, the percentage of oxygen (O_EDX_) from EDX measurements, I_D_/I_G_ is the integrated intensity ratio of the D and G peaks from the Raman spectra, *t* is the average flake thickness obtained from AFM measurements, and *d* is the interlayer spacing corresponding to the (001) reflection of GO obtained from the X-ray diffractograms.
